# Clinical and Etiological Characteristics of Atypical Hand-Foot-and-Mouth Disease in Children from Chongqing, China: A Retrospective Study

**DOI:** 10.1155/2015/802046

**Published:** 2015-11-26

**Authors:** Xiang Yan, Zhen-Zhen Zhang, Zhen-Hua Yang, Chao-Min Zhu, Yun-Ge Hu, Quan-Bo Liu

**Affiliations:** ^1^Department of Infectious Disease, Children's Hospital of Chongqing Medical University, Ministry of Education Key Laboratory of Child Development and Disorders, Chongqing Key Laboratory of Pediatrics, Chongqing 400014, China; ^2^Department of Epidemiology, School of Public Health, University of Michigan, M5124 SPH II, 1415 Washington Heights, Ann Arbor, MI 48109, USA

## Abstract

*Background*. Hand-foot-and-mouth disease (HFMD) is a disease that had similar manifestations to chickenpox, impetigo, and measles, which is easy to misdiagnose and subsequently causes delayed therapy and subsequent epidemic. To date, no study has been conducted to report the clinical and epidemiological characteristics of atypical HFMD.* Methods*. 64 children with atypical HFMD out of 887 HFMD children were recruited, stool was collected, and viral VP1 was detected.* Results*. The atypical HFMD accounted for 7.2% of total HFMD in the same period (64/887) and there were two peaks in its prevalence in nonepidemic seasons. Ten children (15.6%) had manifestations of neurologic involvement, of whom 4 (6.3%) were diagnosed with severe HFMD and 1 with critically severe HFMD, but all recovered smoothly. Onychomadesis and desquamation were found in 14 patients (21.9%) and 15 patients (23.4%), respectively. The most common pathogen was coxsackievirus A6 (CV-A6) which accounted for 67.2%, followed by nontypable enterovirus (26.6%), enterovirus 71 (EV-A71) (4.7%), and coxsackievirus A16 (A16) (1.5%).* Conclusions*. Atypical HFMD has seasonal prevalence. The manifestations of neurologic involvement in atypical HFMD are mild and usually have a good prognosis. CV-A6 is a major pathogen causing atypical HFMD, but not a major pathogen in Chongqing, China.

## 1. Introduction

Hand-foot-and-mouth disease (HFMD) is an infectious disease which mainly affects children under 5 years of age. HFMD is characterized by low-grade fever, general malaise, and vesicles or papulae on the palms, soles, and buttocks.

Since firstly reported in 1957 [1], HFMD began to spread all over the world gradually, which brought heavy economic and social burden to the world. Especially in China, there are several millions of HFMD being reported [2]. Researches showed that enteroviruses were the main pathogens of HFMD, most of which were Enterovirus-A71 (EV-A71) and coxsackievirus A16 (CV-A16) [3, 4]. However, rates of HFMD caused by coxsackievirus A6 (CV-A6) and coxsackievirus A10 have increased by years nowadays in Shenzhen [5], Shandong [6], Guangdong [7], and so on. Besides, there were also other reported pathogens like coxsackievirus A4-5 as well as coxsackievirus B2-5 [8].

In 2008, HFMD characterized by onychomadesis in later phase was first reported in Finland [9], and CV-A6 was the major pathogen. Thereafter, HFMD caused by CV-A6 has been reported in Spain [10], Japan [11], Thailand [12], USA [13], and Singapore [14]. The incidence of CV-A6 related infections was increasing in above countries, and CV-A6 became a major pathogen. Since 2001, CV-A6 has been reported as one of the top 5 pathogens causing HFMD in Taiwan [15], and the incidence of CV-A6-related HFMD increased every year. In addition, in Guangdong China [16], HFMD caused by CV-A6 also has an increased incidence year after year and CV-A6 has been an important pathogen of HFMD. Even an outbreak of HFMD caused by CV-A6 was reported in Changchun, China [17], in 2013. Several studies reported that HFMD caused by CV-A6 was different from previously reported HFMD and characterized by vesicles or papular on the faces, trunks, and limbs or large vesicles or bullae (diameter > 5 mm) on any site of the body, as well as onychomadesis and desquamation in later phase. HFMD with above manifestations is also known as atypical HFMD [18].

We have also encountered atypical HFMD in Chongqing, China, in 2013. This disease had similar manifestations to chickenpox, impetigo, and measles. Thus, it is easy to be misdiagnosed, subsequently causing delayed therapy and subsequent epidemic. To date, no study has been conducted to report the clinical and epidemiological characteristics of atypical HFMD. Chongqing is a southwestern city of China with a large population, a relatively poor sanitary condition, and a high incidence of infectious diseases. Thus, we should pay more attention to the prevention of HFMD. This study aimed to summarize the epidemiological, clinical, and etiological characteristics of atypical HFMD diagnosed between September 2013 and August 2014 in Chongqing, China, which may be helpful for the diagnosis, differential diagnosis, and therapy of HFMD.

## 2. Material and Methods

### 2.1. Patients

A total of 887 hospitalized children were diagnosed with HFMD in the Children's Hospital of Chongqing University between September 2013 and August 2014. Of these patients, 64 children (7.2%, 64/887) with atypical HFMD were recruited into this study for further analysis. Informed consent was obtained. This study has been approved by the Ethics Committee related to the Children's Hospital of Chongqing University.

### 2.2. Inclusion Criteria

HFMD patients with following characteristics were recruited into present study. Characteristics of atypical rashes were as follows: (1) distribution: papules and/or vesicles (diameter < 5 mm) being also found in other sites such as face, neck, perioral area, trunk, limbs, and externalia besides the palms, soles, and buttocks; and/or (2) morphology of rashes: large vesicles or bullae (diameter > 5 mm), erosive lesions, purpuric/petechial lesions, or “eczema coxsackium” at any site [18, 19]. Detection of enterovirus was positive or serological detection was positive. The clinical diagnosis, etiological diagnosis, and diagnosis of common HFMD and severe HFMD were performed according to the Guideline for the Clinical Diagnosis and Therapy of Hand-Foot-Mouth Disease developed by Chinese Ministry of Health in 2010. Fever was defined as body temperature of ≥37.5°C; onychomadesis was defined when a nail separated from the nail bed (detach) due to the stop of transverse ridge growth or transverse ridge mutilation after long term restriction of parent material activity.

### 2.3. Exclusion Criteria

(1) Informed consent was not obtained from their parents or their parents withdraw from this study before the end of study; (2) stools were not collected, or the clinical information was incomplete; (3) there were skin lesions caused by other diseases (such as chickenpox, impetigo, and measles).

### 2.4. Sample Collection

This study was approved by the Ethics Committee of Children's Hospital of Chongqing University (number: 105/2014). Informed consent was obtained from parents of participants before study. Stools were collected from 64 children with atypical HFMD in acute phase (within 3 days after disease onset) and stored at −80°C for further detection. In addition, the rashes were photographed, and onychomadesis, desquamation, and sequelae were closely monitored during follow-up period. The patients' characteristics and clinical manifestations were collected from the medical record. At the same time, the characteristics of all the patients with different types of HFMD were also recorded in the same period for the epidemiological comparisons.

### 2.5. Virus Detection and DNA Sequencing

RNA was extracted from stool samples by using QIAampH MinElute Virus Spin Kits (Qiagen, Hilden, Germany) and cDNA was synthesized by using Reverse Transcription System A3500 (Promega, Madison, WI, USA) according to the manufacturer's instructions. Nested-PCR was performed to detect universal enteroviruses (EV-U), EV-A71, CV-A16, respectively, while ordinary PCR was performed to detect CV-A6. Primers for CV-A6 were 5′-TGGTAGGAGTTGTGGAGGT-3′ (forward) and 5′-CCTTCATAATCYGTAGTGGTT-3′ (reverse), which was constructed by ourselves. And primers for other genes were obtained from the study of Ge et al. [20]. Sequencing was performed in 43 samples positive for CV-A6, and results were compared with those in BLAST (www.ncbi.nlm.nih.gov/blast). Neighbor-joining (NJ) method was employed to construct phylogenetic tree with bootstrap method 1000 as a parameter.

### 2.6. Statistical Analysis

Data with normal distribution were expressed as mean ± standard deviation ($\overset{-}{x}\pm s$). Data with abnormal distribution were presented as range (M). Statistical analysis was performed with SPSS version 17.0 (SPSS Inc., Chicago, IL, USA). *P* < 0.05 was considered statistically significant.

## 3. Results

### 3.1. Incidence of Atypical HFMD in Chongqing

**Figure 1:**
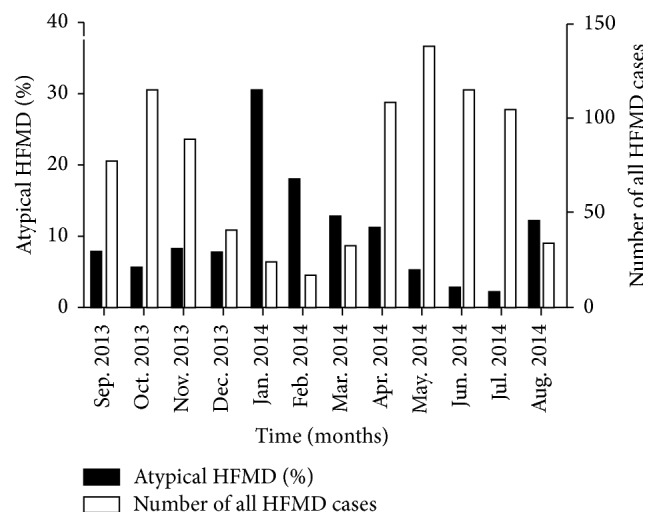
Proportion of atypical HFMD to total HFMD per month and number of total HFMD in each month. Black: proportion of atypical HFMD; white: number of total HFMD. *X*-axis: time; left *Y*-axis: proportion of atypical HFMD; right *Y*-axis: number of total HFMD.

During this study, a total of 887 hospitalized children were diagnosed with HFMD, of whom 7.2% (64/887) were atypical HFMD. Both total and atypical HFMD had two peaks in their incidences. The proportion of atypical HFMD varied over time and ranged from 1.9% to 30.4%. The proportion was the highest from January to March, a season transiting from winter to spring, in which atypical HFMD accounted for 30.4% (7/23), 17.6% (3/17), and 12.5% (4/32), respectively. From January to March, the highest proportion of atypical HFMD was found in January. Another peak was found from August to October in which the proportion of atypical HFMD was 12.1% (4/33), 7.8% (6/77), and 5.3% (6/114), respectively. Besides, atypical HFMD was also found in other seasons. However, total HFMD peaked from April to July, a season transiting from spring to summer, in which total HFMD accounted for about 52.2% in the study period (443/887). The second peak was found from September to November, a season transiting from autumn to winter, and total HFMD in these months accounted for 31.5% in the study period (279/887) (Figure 1).

### 3.2. Demographic Characteristics of Atypical HFMD

Among the 64 children with atypical HFMD, there were 40 males and 24 females with the male to female ratio of 1.67 : 1. The age of children ranged from 6 months to 48 months (median: 15 months). Atypical HFMD mainly occurred in children younger than 3 years, which accounted for 93.8% (60/64), and 54.7% (35/64) of children were younger than 1 year. Most patients lived in the city (62.5%, 40/64). A majority of children lived scattered (79.7%, 51/64) and remaining children were on nursery care (15.6%, 10/64) and schooling (4.7%, 3/64) (Table 1).

### 3.3. Clinical Manifestations

Atypical HFMD children usually presented with fever (79.7%, 51/64), poor appetite (67.2%, 43/64), and salivation (64.1%, 41/64). In addition, 10 children had manifestations of neurologic involvement of whom the startle response (15.6%, 10/64), vomiting (7.8%, 5/64), and convulsion (12.5%, 8/64) had higher prevalences, but headache and limb trembling were found in only 2 patients, unconsciousness in 1 and unsteady gait in 1. Severe atypical HFMD was observed in 5 patients (7.8%, 5/64), of whom 4 had severe atypical HFMD (6.3%, 4/64) and 1 had critically severe atypical HFMD (1.5%, 1/64), but all these children recovered smoothly after therapy without any sequela (Table 1).

### 3.4. Characteristics of Rashes

**Figure 2:**
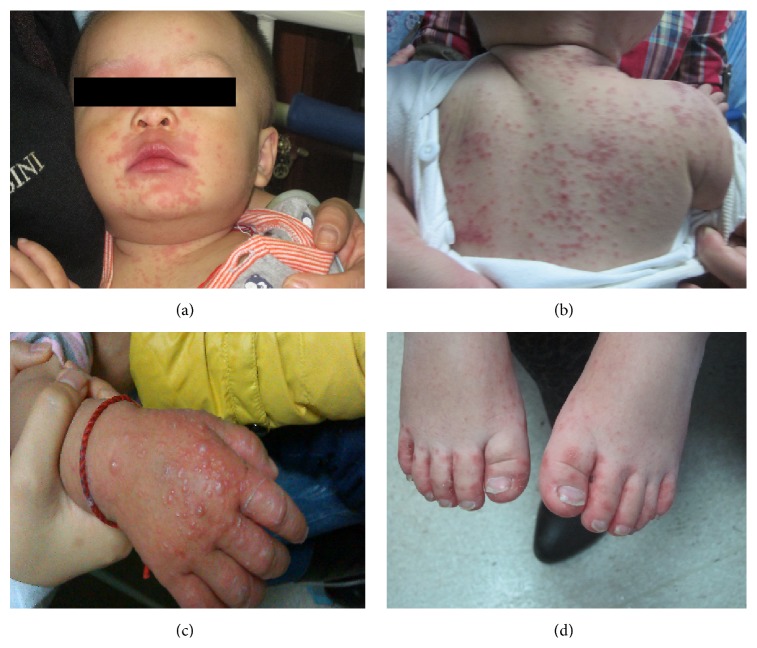
Characteristics of rashes in atypical HFMD. (a) A boy aged 1 year, and papulae were mainly distributed on the perioral area and face; (b) a boy aged 1 year and 1 month, and papulae/vesicles were found at the back; (c) a girl aged 1 year and 6 months, and papulae were found on the hand; (d) a boy aged 1 year and 8 months, and erosions were noted in both feet.

Rashes of atypical HFMD were distributed not only in typical sites but on other sites (such as lower limbs [36/64, 56.3%], face [34/64, 53.1%], trunk [27/64, 42.2%], upper limb [19/64, 29.7%], and externalia [9/64, 14.1%]). Rashes on lower limbs were mainly found in the thigh (17/36, 47.2%) and those on face were mainly noted in perioral area (20/34, 58.8%). In respect of the number of sites with involvement, 5–7 (59.4%, 38/64) sites were the most common, followed by 2–4 (31.3%, 20/64) and 8-9 (9.3%, 6/64). The maximum number of sites with involvement was 9. In respect of rash morphology, papula-dominant rashes were found in 44 patients (68.8%, 44/64) and vesicle-dominant rashes in 41 children (64.1%, 41/64), 22 children had both papula and vesicle (34.4%, 22/64), 13 patients (20.3%, 13/64) showed large vesicles, 2 had erosive lesions (3.1%, 2/64) with itching, and 3 (4.7%, 3/64) had scabs in late phase (Figure 2 and Table 2).

### 3.5. Complications in Late Phase: Onychomadesis and Desquamation

**Figure 3:**
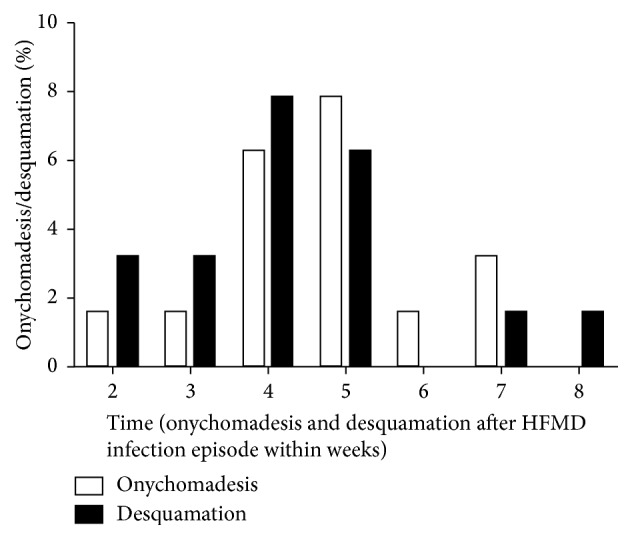
Incidences of onychomadesis and desquamation in atypical HFMD children at different time points. *X*-axis: time of follow-up after acute phase; *Y*-axis: proportion of children with onychomadesis/desquamation in children receiving follow-up. Statistical analysis was performed with SPSS version 17.0 (SPSS Inc., Chicago, IL, USA). A value of *P* < 0.05 was considered statistically significant.

All the children received follow-up after therapy. Of these patients, 14 (21.9%, 14/64) developed onychomadesis and 15 (23.4%, 15/64) had desquamation. In 14 children with onychomadesis, the most common virus in children with onychomadesis was CV-A6 (71.4%, 10/14), followed by nontypable enterovirus (14.3%, 2/14) and EV-A71 (14.3%, 2/14). The mean time to onychomadesis was 4.7 weeks after the acute phase of HFMD (range: 2–7 weeks), and onychomadesis was usually found at 5th week (5/14, 35.7%). Onychomadesis was mainly found in the right thumb (8/14, 57.1%) and with a mean of 4.7 fingers/toes (range: 1–20). The most common virus in children with desquamation was CV-A6 (80%; 12/15), followed by nontypable enterovirus (13.3%, 2/15) and EV-A71 (6.7%; 1/15). The mean time to desquamation was 4.3 weeks after the acute phase of HFMD (range: 2–8 weeks) and desquamation was usually found at 4th week (5/15, 33.3%). Of note, desquamation was often found at the sites with rashes (12/15, 80.0%) (Table 2 and Figure 3).

### 3.6. Etiological Characteristics of Atypical HFMD and DNA Sequencing

**Figure 4:**
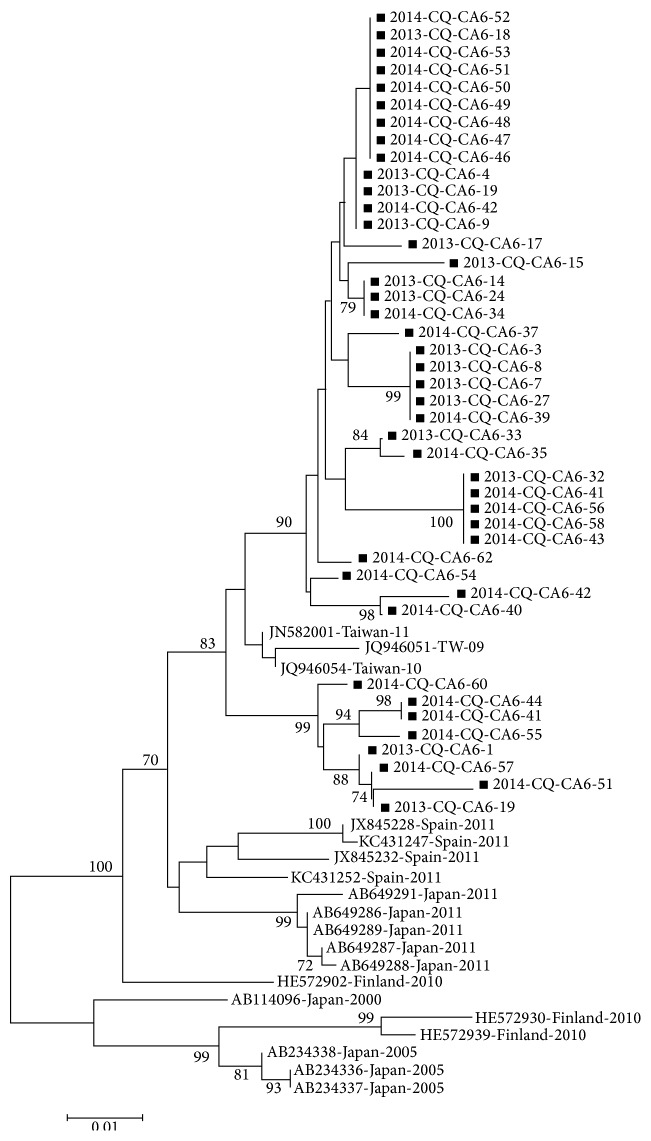
Phylogenetic analysis of coxsackievirus A6 strains based on the partial VP1 gene sequence (nucleotide position: 254–864). ■: virus separated in the present study, and remaining viruses were from Genbank including 2011 Spain strains (JX845228, JX845232, KC431247, and KC431252), 2000–2011 Japan strains (AB114096, AB234336, AB234337, AB234338, AB649286, AB649287, AB649288, AB649289, and AB649291), 2010 Finland strains (HE572902, HE572930, and HE572939), and 2009–2011 Taiwan strains (JN582001, JQ946051, and JQ946054).

Among these 64 patients with atypical HFMD, RT-nested PCR showed all the samples were positive for EV-U, and the most common pathogen was CV-A6 (67.2%; 43/64), followed by nontypable enterovirus, EV-A71, and CV-A16 which were found in 26.6% (17/64), 4.7% (3/64), and 1.5% (1/64) of patients, respectively. Furthermore, sequencing was performed in 43 samples positive for CV-A6 and the fragment was 630 bp in length. After homology analysis of nucleotides and amino acids, results showed the homology of CV-A6 was 87.6%–100% and 94.5%–100% in the nucleotides and amino acids, respectively, as compared with the strain found in Spain, Finland, Japan, and Taiwan (Figure 4).

## 4. Discussion

The clinical characteristics of atypical HFMD are usually atypical, which brings difficulty to the clinical diagnosis and therapy of HFMD, which may cause misdiagnosis and delayed therapy, resulting in regional or large-scale epidemic. To date, no study has been conducted to investigate the epidemiological and clinical characteristics of atypical HFMD. In the present study, we for the first time described the epidemiological, clinical, and etiological characteristics of atypical HFMD in Chongqing, China, between September 2013 and August 2014. Our findings may provide significant evidence for the clinical diagnosis, timely isolation, and therapy of atypical HFMD.

Results of this study showed that atypical HFMD had obviously seasonal prevalence, was often found in young children and in urban children, and usually predicted a good prognosis. In atypical HFMD children, rashes were widely distributed, skin lesions were severe, and the incidences of onychomadesis and desquamation were relatively high in late phase of atypical HFMD. Further etiological analysis showed CV-A6 was a major pathogen causing atypical HFMD, which had a high homology to the strain found in Finland, Taiwan, and other regions.

In the study period, the prevalence of atypical HFMD showed seasonal characteristics, and its incidence varied in different seasons. The incidence of atypical HFMD was high in the seasons transiting from winter to spring and from summer to autumn, but that of total HFMD was high in the seasons transiting from spring to summer and from autumn to winter. That is, the peak of atypical HFMD occurred in the troughs of total HFMD, but that of total HFMD occurred in the troughs of atypical HFMD. It is well known that the pathogenesis of HFMD is closely related to climatic factors such as temperature, humidity, and rainfall. The higher the humidity and temperature, the higher the possibility the presence of HFMD has within a certain range [21]. Previous studies [22, 23] have shown that HFMD peaks in the season transiting from spring to summer, followed by the season transiting from autumn to winter, which is consistent with our findings. In Chongqing, China, the peaks of atypical HFMD occurred alternatively with the total HFMD. This was not consistent with the findings that the peak of total HFMD overlaps that of atypical HFMD in Taiwan and Japan [18, 24]. However, whether the characteristics of atypical HFMD were related to the geographical region, climate, race, and survival conditions of CV-A6 (optimal temperature, humidity, and wind velocity for CV-A6 growth) and whether there was lack of temperature sensitive amino acids [25] are still unclear, and the specific mechanisms are required to conduct further investigations. This also reminds us that it is necessary to prevent HFMD in the HFMD prevalent seasons and also pay attention to the seasons of atypical HFMD to prevent regional or large scale epidemic.

In our study, atypical HFMD was mainly found in children younger than 3 years old, and more than 50% children (54.7%) were younger than 1 year old. This was similar to findings in the study of Puenpa et al. [12]. Whether the young age is associated with special features of CV-A6 or mutation of CV-A6 in atypical HFMD is still poorly understood, but these findings indicate the correlation between atypical HFMD and young age. This reminds us that we should emphasize the prevention and monitoring of atypical HFMD in children younger than 1 year.

In the present study, only 15.6% of children with atypical HFMD had manifestations of neurologic involvement. Further analysis showed 4 cases were severe HFMD and 1 was critically severe HFMD, but all of these children recovered smoothly after therapy without sequelae, suggesting a good prognosis. This was in agreement with findings reported by Huang et al. [18]. This indicates that atypical HFMD has a good prognosis but still has a possibility to cause severe HFMD, or even critically severe HFMD, and clinicians should pay attention to atypical HFMD. Few studies have been conducted to report the clinical characteristics of atypical HFMD and more studies are required to investigate the prognosis of atypical HFMD.

In children with atypical HFMD, the rashes were widely distributed and were diverse in morphology, which were in accordance with previously reported data in USA and Japan [13, 19, 24]. Thus, atypical HFMD is easy to misdiagnose as chickenpox, impetigo, and measles, which may delay its diagnosis and therapy. Rashes were also found on the face, trunk, and limbs besides palms, soles, and buttocks, 64.1% of children developed vesicles, and some children also had scabs in late phase. These manifestations were in agreement with HFMD caused by CV-A6 in Japan [24]. This suggests that clinicians should notice the atypical rashes, especially the vesicles-dominant rashes that cannot be explained by other diseases, because these might be manifestations of atypical HFMD, and pathogen detection of the stool is recommended.

In late phase, the incidences of onychomadesis and desquamation were relatively high. A lot of pathogens may cause onychomadesis and desquamation, but CV-A6 is a major pathogen, which was in accordance with findings reported in Taiwan [26]. Onychomadesis and desquamation occurred mainly at 4-5 weeks after the acute phase, which was in agreement with the time point reported by Österback et al. [9]. In addition, Österback et al. found CV-A6 was also detected in the shedding nails and thus they proposed that viral replication caused the destruction of nail matrix. However, Shikuma et al. [27] found onychomadesis occurred only in the fingers with severe skin lesions, which might be ascribed to the severe inflammation after skin injury. Whether onychomadesis is related to viral replication or inflammation or ascribed to the overprevention and overprotection (such as repeated hand-washing) [28] is still unclear, and further investigation is required.

In early studies on the etiology of HFMD, results showed EV-A71 and CV-A16 were the major pathogens [3, 4], and HFMD caused by EV-A71 and CV-A16 was prevalent alternatively in different regions. Since 2008, the incidence of HFMD caused by CV-A6 has been increasing in Southeast Asia and Europe [9–14]. In the present study, more than 65% of atypical HFMD was caused by CV-A6, which was consistent with that reported in Taiwan in 2010 [18]. These findings highlight the importance of CV-A6 in the pathogenesis of atypical HFMD. However, CV-A6 is not a common strain in Chongqing, and whether the CV-A6 increases over year in Chongqing is required to be further confirmed in future studies.

There were still limitations in the present study. First, not all the children with atypical HFMD were hospitalized for therapy, and thus the sample size of atypical children was still small. The clinical features collected from the hospitalized children with atypical HFMD might not represent the characteristics of all the children with atypical HFMD. In addition, some parents might neglect the onychomadesis and desquamation in late phase, which may underestimate the incidence of complications.

Taken together, the atypical HFMD has an obviously seasonal prevalence, special clinical manifestations, and a relatively good prognosis. Rashes in atypical HFMD are widely distributed and are diverse in morphology, the incidences of onychomadesis and desquamation are relatively high in late phase, and atypical HFMD is mainly caused by CV-A6 in Chongqing, China.

## Figures and Tables

**Table 1:** Demographic and clinical presentations of patients with atypical HFMD.

Variables	Atypical HFMD (*n* = 64) *n* (%)	95% CI
*Demographics*		
Age (years)		
≤1	35 (54.7)	53.7%~55.7%
1–3	25 (39.1)	37.9%~40.3%
>3	4 (6.2)	5.5%~6.9%
Sex		
Male	40 (62.5)	61.6%~63.4%
Female	24 (37.5)	36.3%~38.7%
Residence		
Urban	40 (62.5)	61.6%~63.4%
Rural	24 (37.5)	36.3%~38.7%
Children care		
Home care	51 (79.7)	79.1%~80.3%
Nursery care	10 (15.6)	14.6%~16.6%
Schooling	3 (4.7)	4.1%~5.3%
*Symptoms*		
Fever		
≥37.5°C	51 (79.7)	79.1%~80.3%
<37.5°C	13 (20.3)	19.2%~21.4%
Cough		
Yes	20 (31.3)	30.1%~32.5%
No	44 (68.7)	67.9%~69.5%
Rhinorrhea		
Yes	15 (23.4)	22.3%~24.5%
No	49 (76.6)	76.0%~77.2%
Salivation		
Yes	41 (64.1)	63.2%~65.0%
No	23 (35.9)	34.7%~37.1%
Diarrhea		
Yes	9 (14.1)	13.1%~15.1%
No	55 (85.9)	85.5%~86.3%
Poor oral intake		
Yes	43 (67.2)	66.4%~68.0%
No	21 (32.8)	31.6%~34.0%
Vomiting		
Yes	5 (7.8)	7.0%~8.6%
No	59 (92.2)	92.0%~92.4%
Headache		
Yes	2 (3.1)	25.8%~36.2%
No	62 (96.9)	96.8%~97.0%
Febrile seizure		
Yes	8 (12.5)	11.6%~13.4%
No	56 (87.5)	87.1%~87.9%
Startle response		
Yes	10 (15.6)	14.6%~16.6%
No	54 (84.4)	84.0%~84.8%
Altered consciousness		
Yes	1 (1.6)	1.2%~2.0%
No	63 (98.4)	98.3%~98.4%
Limb trembling		
Yes	2 (3.1)	2.6%~3.6%
No	62 (96.9)	96.8%~97.0%
Unsteady gait		
Yes	1 (1.6)	1.2%~2.0%
No	63 (98.4)	98.3%~98.4%

HFMD, hand-foot-and-mouth disease. Statistical analysis was performed with SPSS version 17.0 (SPSS Inc., Chicago, IL, USA). A value of *P* < 0.05 was considered statistically significant.

**Table 2:** Rashes and complications of patients with atypical HFMD.

Variables	Atypical HFMD (*n* = 64) *n* (%)	95% CI
*Rash distribution*		
Palm/soles		
Yes	51 (79.7)	79.1%~80.3%
No	13 (20.3)	19.2%~21.4%
Oral ulcer		
Yes	47 (73.4)	72.7%~74.1%
No	17 (26.6)	25.4%~27.8%
Face		
Yes	34 (53.1)	52.1%~54.1%
No	30 (46.9)	45.8%~48.0%
Torso		
Yes	27 (42.2)	41.1%~43.3%
No	37 (57.8)	56.8%~58.8%
Arms		
Yes	19 (29.7)	28.5%~30.9%
No	45 (70.3)	69.5%~71.1%
Legs		
Yes	36 (56.3)	55.3%~57.3%
No	28 (43.7)	42.6%~44.8%
Buttocks		
Yes	50 (78.1)	77.5%~78.7%
No	14 (21.9)	20.8%~23.0%
Externalia		
Yes	9 (14.1)	13.1%~15.1%
No	55 (85.9)	85.5%~86.3%
*Morphology*		
Vesicle		
Yes	41 (64.1)	63.2%~65.0%
No	23 (35.9)	34.7%~37.1%
Papula		
Yes	44 (68.8)	68.0%~69.6%
No	20 (31.2)	30.0%~32.4%
Bulla		
Yes	13 (20.3)	19.2%~21.4%
No	51 (79.7)	79.1%~80.3%
Erosions		
Yes	2 (3.1)	2.6%~3.6%
No	62 (96.9)	96.8%~97.0%
*Long-term complications*		
Desquamation		
Yes	15 (23.4)	22.6%~24.5%
No	49 (76.6)	76.0%~77.2%
Onychomadesis		
Yes	14 (21.9)	20.8%~23.0%
No	50 (78.1)	77.5%~78.7%

HFMD, hand-foot-and-mouth disease. Statistical analysis was performed with SPSS version 17.0 (SPSS Inc., Chicago, IL, USA). A value of *P* < 0.05 was considered statistically significant.
